# HPV E6 and E7 mRNA Test for the Detection of High-Grade Cervical Lesions

**DOI:** 10.1001/jamanetworkopen.2024.59698

**Published:** 2025-02-11

**Authors:** Awoke Derbie, Melanie Maier, Bereket Amare, Eyaya Misgan, Endalkachew Nibret, Fantahun Biadglegne, Uwe G. Liebert, Tsion Zewdu Minas, Muluken Agage Yenesew, Dabere Nigatu, Tesfaye B. Mersha, Daniel A. Enquobahrie, Yimtubezinash Woldeamanuel, Tamrat Abebe

**Affiliations:** 1 College of Medicine and Health Sciences, Bahir Dar University, Bahir Dar, Ethiopia; 2 Centre for Innovative Drug Development and Therapeutic Trials for Africa, College of Health Sciences, Addis Ababa University, Addis Ababa, Ethiopia; 3 Department of Virology, Institute of Medical Microbiology and Virology, Leipzig University Hospital, Leipzig, Germany; 4 Department of Biology, College of Science, Bahir Dar University, Bahir Dar, Ethiopia; 5 Interventional Radiology Innovation at Stanford, Department of Radiology, Stanford University School of Medicine, Palo Alto, California; 6 Department of Pathology, Johns Hopkins School of Medicine, Baltimore, Maryland; 7 Department of Pediatrics, Cincinnati Children’s Hospital Medical Center, Cincinnati, Ohio; 8 School of Public Health, Department of Epidemiology, University of Washington, Seattle; 9 School of Medicine, College of Health Sciences, Addis Ababa University, Addis Ababa, Ethiopia

## Abstract

This cross-sectional study examines whether human papillomavirus (HPV) messenger RNA (mRNA)–based testing can identify women with high-risk abnormal cervical lesions..

## Introduction

Cervical cancer (CC) is a common public health problem worldwide. Over 95% of CC cases are associated with genital infection with high-risk human papillomavirus (HR-HPV).^1^ CC screening and diagnosis strategies vary across countries. Although the most frequent screening method is cytological testing, there are alternative techniques. HPV E6 and E7 messenger RNA (mRNA) are promising noninvasive biomarkers in testing high-grade cervical intraepithelial neoplasia grade 2 or higher (CIN2+) that allows detection of HPV infection and estimation of change in cervical lesions.^2^ Therefore, we aimed to assess the diagnostic effectiveness of the E6 and E7 mRNA test for detecting CIN2+ among Ethiopian women with gynecological concerns.

## Methods

This hospital-based cross-sectional study was conducted at Felege Hiwot Comprehensive Specialized Hospital in northwest Ethiopia between March 1, 2019, and October 30, 2021. The Addis Ababa University Institutional Review Board and the Ethiopian National Research Ethics Review Committee approved the study. Written informed consent was provided by participants. We followed the STROBE reporting guideline.

Using consecutive sampling techniques, cervical specimens were collected from women who presented with signs and symptoms suggestive of abnormal cervical lesions. A gynecologist collected punch biopsies for histopathologic examinations and cervical swabs for HPV DNA and E6 and E7 mRNA testing (eMethods in Supplement 1). Data were analyzed from month year to month year with Stata, version 14.1 (StataCorp LLC).

## Results

Among 355 participants (mean [SD] age, 46.4 [11.4] years), the prevalence of HR-HPV was 53.0% (188). Of 335 participants with cervical histological examination, 140 (41.8%) were diagnosed with CC. CIN2+ (including CIN2, CIN3, and cancer) was detected in 164 participants (49.0%). One hundred twenty-seven participants (35.8%) tested positive for HPV-16, HPV-18, and HPV-45 using the E6 and E7 mRNA test. Positivity rate of the HPV DNA test for these 3 HPVs was 42.0% (149). The mRNA test positivity rate increased as the cervical lesion grade increased: 5.0% (2 of 40) in CIN1, 25.0% (3 of 12) in CIN2, 50.0% (6 of 12) in CIN3, and 70.1% (94 of 134) in CC (Table 1).

**Table 1.** Human Papillomavirus E6 and E7 mRNA Test Distribution by Cervical Histopathologic Grades in Northwest Ethiopia, 2021^a^

E6 and E7 mRNA test	Histopathologic grades, No. (%)					Total
	Normal	CIN1	CIN2	CIN3	Cancer	
HPV-16	8 (11.0)	2 (5.0)	2 (16.7)	6 (50.0)	80 (59.7)	98 (36.2)
HPV-18	0	0	1 (8.3)	0	8 (6.0)	9 (3.3)
HPV-45	0	0	0	0	4 (3.0)	4 (1.5)
HPV-16 and HPV-45	0	0	0	0	2 (1.5)	2 (0.7)
mRNA not detected	65 (89.0)	38 (95.0)	9 (75.0)	6 (50.0)	40 (29.9)	113 (41.7)
Total	73	40	12	12	134	271

Abbreviations: CIN, cervical intraepithelial neoplasia; HPV, human papillomavirus.

^a^ Histological reports for cervicitis (n = 30), cervical polyp (n = 10), and other conditions (not otherwise specified; n = 17) that include unremarkable endocervical tissues; myoma; and cervical wart, among others, are not included in this table to make the data more precise.

Sensitivity and specificity of the mRNA assay for detecting CIN2+ were 65.2% (95% CI, 57.5%-72.2%) and 90.0% (95% CI, 84.6%-93.4%), respectively. The HPV DNA test’s detection sensitivity was higher at 84.8% (95% CI, 78.4%-89.6%), but its specificity was lower at 74.1% (95% CI, 67.1%-80.1%) (Table 2). Sensitivity, specificity, positive predictive value, and negative predictive value of the mRNA test for detecting CIN2+ among HPV DNA test–positive cases were 92.7% (95% CI, 86.2%-96.2%), 47.0% (95% CI, 30.9%-63.6%), 85.6% (95% CI, 78.1%-90.8%), and 65.2% (95% CI, 44.9%-81.2%), respectively.

**Table 2.** Human Papillomavirus DNA and E6 and E7 mRNA Tests for Detecting Histologically Confirmed CIN2+ in Northwest Ethiopia, 2021

Test	CIN2+, No. (%) [n = 328]		% (95% CI)			
	Yes	No	Sensitivity	Specificity	PPV	NPV
HPV DNA						
Positive	134 (40.9)	44 (13.4)	84.8 (78.4-89.6)	74.1 (67.1-80.1)	75.3 (68.5-81.0)	84.0 (77.3-89.0)
Negative	24 (7.3)	126 (38.4)				
HPV E6 and E7 mRNA						
Positive	103 (31.4)	17 (5.2)	65.2 (57.5-72.2)	90.0 (84.6-93.4)	85.8 (78.5-91.0)	73.6 (67.2-79.1)
Negative	55 (16.8)	153 (46.6)				

Abbreviations: CIN2+, cervical intraepithelial neoplasia 2 or 3 or cancer; HPV, human papillomavirus; NPV, negative predictive value; PPV, positive predictive value.

## Discussion

The positivity rate of the E6 and E7 mRNA test increasing with higher histopathologic grade implies that the presence of E6 and E7 mRNA transcripts is important in cervical disease progression. The study supports the hypothesis that E6 and E7 mRNA is a potential marker for women at increased risk for developing CC, as the test is associated with severity of cervical lesions compared with the HPV DNA test.^3^ Most HPV infections can resolve on their own after several years. Only actively infected cells with HR-HPVs produce E6 and E7 mRNA, and this expression increases as CIN develops and progresses.^4^ Among mRNA test–positive cases, HPV-16 was the most predominant in expressing mRNA across different histopathologic grades, followed by HPV-18 and HPV-45.

High specificity of the mRNA test is partly explained by the test targeting only 3 HPV genotypes. However, studies in the field assert that the mRNA test has good specificity for CIN2+ detection.^5,6^

A study limitation is that, due to financial constraints, the mRNA analysis did not incorporate all relevant HR-HPVs identified by the HPV DNA test. The findings imply that the mRNA test can serve as a better triage test to reduce colposcopy and biopsy referral and reduce overtreatment and adverse psychological consequences for Ethiopian women.

## Notes

Supplement 1.
eMethods.

Supplement 2.
Data Sharing Statement

## References

[1] Leto Md, Santos Júnior GF, Porro AM, Tomimori J. Human papillomavirus infection: etiopathogenesis, molecular biology and clinical manifestations. An Bras Dermatol. 2011;86(2):306-317. doi:10.1590/S0365-05962011000200014 21603814

[2] Duvlis S, Popovska-Jankovic K, Arsova ZS, Memeti S, Popeska Z, Plaseska-Karanfilska D. HPV E6/E7 mRNA versus HPV DNA biomarker in cervical cancer screening of a group of Macedonian women. J Med Virol. 2015;87(9):1578-1586. doi:10.1002/jmv.24199 25880030

[3] Zhang SK, Guo Z, Wang P, . The potential benefits of HPV E6/E7 mRNA test in cervical cancer screening in China. Front Oncol. 2020;10:533253. doi:10.3389/fonc.2020.533253 33123463 PMC7567165

[4] Fontecha N, Basaras M, Hernáez S, Andía D, Cisterna R. Assessment of human papillomavirus E6/E7 oncogene expression as cervical disease biomarker. BMC Cancer. 2016;16(1):852. doi:10.1186/s12885-016-2885-x 27816058 PMC5097850

[5] Derbie A, Mekonnen D, Woldeamanuel Y, Van Ostade X, Abebe T. HPV E6/E7 mRNA test for the detection of high grade cervical intraepithelial neoplasia (CIN2+): a systematic review. Infect Agent Cancer. 2020;15:9-9. doi:10.1186/s13027-020-0278-x 32047531 PMC7006188

[6] Dabeski D, Duvlis S, Basheska N, . Comparison between HPV DNA testing and HPV E6/E7 MRNA testing in women with squamous cell abnormalities of the uterine cervix. Pril (Makedon Akad Nauk Umet Odd Med Nauki). 2019;40(1):51-58. doi:10.2478/prilozi-2019-0003 31152639

