# Supplementary material for: HPV E6 and E7 mRNA Test for the Detection of High-Grade Cervical Lesions
Source: JAMA Netw Open. 2025 Feb 11;8(2):e2459698. doi: 10.1001/jamanetworkopen.2024.59698 (PMC11815524; doi:10.1001/jamanetworkopen.2024.59698)
Supplement: Supplement 1. — eMethods. [file jamanetwopen-e2459698-s001.pdf]

Supplemental Online Content

Derbie A, Maier M, Amare B, et al. HPV E6 and E7 mRNA test for the detection of high-grade cervical lesions. *JAMA Netw Open*. 2025;8(2):e2459698. doi:10.1001/jamanetworkopen.2024.59698

eMethods

This supplemental material has been provided by the authors to give readers additional information about their work.

© 2025 Derbie A et al. *JAMA Network Open*

eMethods

HPV E6/E7 mRNA Extraction

Frozen cervical swabs were thawed from the freezer and relabeled with a sequential barcode. Then each tube was vortexed (3-5 seconds) to homogenize the cells located on the brush with the preservative solution. In some samples with visible blood clots up to 400µl phosphate buffer solution (PBS) was added before vortexing. The brush was removed after the cells were released into the preservative solution. For the E6/E7-mRNA PCR, 200 µl of the sample was extracted using the DNA and Viral NA Small Volume Kit on a MagNA Pure 96 instrument (Roche, Mannheim, Germany) as per the manufacturer instructions with elution volume set to 50 µl [37]. The remaining vortexed sample in the original sample collection tube was used for HPV DNA detection and genotyping on the Alinity m System (Abbott Molecular, Des Plaines, IL, USA).

HPV DNA detection and genotyping

HR-HPV detection and characterization were made using the Alinity m HR HPV AMP Kit on Alinity m System (software version: 1.6.3) (Abbott Molecular, Des Plaines, IL, USA) at the Institute of Virology, Leipzig University Hospital, Germany.

HPV E6/E7 mRNA Assay

Due to time and resource constraints, the E6/E7 mRNA multiplex assay was reduced to detect HPV16, 18 and 45 genotypes. Primers for corresponding HPV genotypes were used according to the previously published primers and probes, (Metabion, Germany) [43]. The following pair of E6/E7 region primers of these HPVs (Table 1) were used in a duplex format for the mRNA detection and the reaction and amplification conditions were performed as per the in-house protocols of the Institute of Virology, Leipzig University Hospital, Germany. Similarly, corresponding probes from the publication were modified with for higher stability (TibMolbiol, Germany).

Type of primers and probes used for the detection of HR-HPV E6/E7 mRNAs.

Primers		
**HPV type**	**Forward**	**Reverse**
HPV16	F 5’- GAGATACACCTACATTGCATGA-3	HPV16_R 5’- GCTGGACCATCTATTTCATCCTC-3

© 2025 Derbie A et al. *JAMA Network Open*

HPV18	F 5’- CATTGTATTGCATTTAGAGCCC-3	HPV18_R 5’- GCTACTACTAGCTCAATTCTGGC-3
HPV45	F 5’- GTAGGGAAACACAAGTATAGCA-3	HPV45_R 5’-CAACAGGTCAACAGGATCTAAT-3
**Probes**		
HPV16	HPV16P_LNAFAM 5’- 6FAM-TgCAACCAgAg+AC+A+ACT-BHQ1-3’	
HPV18	HPV18P_LNAFAM 5’- 6FAM-TCCggTTgACCTTCT+AT+gT -BHQ1-3’	
HPV45	HPV45P_LNAFAM 5’- 6FAM-TCCggTTgACCTTCT+AT+gTAAgTATgCATggA+C+CCC -BHQ1-3’	

The RT-PCR was performed using the SuperScript III Platinum One-Step qRT-PCR Kit based on the manufacturer’s instructions.

Ethics: The study protocol was approved by the institutional review board (IRB) of the College of Health Sciences, Addis Ababa University (087/19/DMIP) and by the Ethiopian National Research Ethics Review Committee (7/2-149/m259/35).

© 2025 Derbie A et al. *JAMA Network Open*
