# Supplementary material for: HPV E6 and E7 mRNA Test for the Detection of High-Grade Cervical Lesions
Source: JAMA Netw Open. 2025 Feb 11;8(2):e2459698. doi: 10.1001/jamanetworkopen.2024.59698 (PMC11815524; doi:10.1001/jamanetworkopen.2024.59698)
Supplement: Supplement 2. — Data Sharing Statement [file jamanetwopen-e2459698-s002.pdf]

Data Sharing Statement

Derbie. HPV E6 and E7 mRNA Test for the Detection of High-Grade Cervical Lesions. *JAMA Netw Open*. Published February 11, 2025. doi:10.1001/jamanetworkopen.2024.59698

Data
Data available: No

Additional Information
Explanation for why data not available: The data will be available from the corresponding author upon request.
